# The Mediatory Role of the Boredom and Loneliness Dimensions in the Development of Problematic Internet Use

**DOI:** 10.3390/ijerph20054446

**Published:** 2023-03-02

**Authors:** Laura Orsolini, Giulio Longo, Umberto Volpe

**Affiliations:** Unit of Clinical Psychiatry, Department of Clinical Neurosciences/DIMSC, Polytechnic University of Marche, 60126 Ancona, Italy

**Keywords:** boredom, loneliness, problematic Internet use, PIU, youth, anxiety, depression

## Abstract

In recent years, there has been a gradual digitalization of our society, resulting in intensified technology use for daily life activities, including the emergence of problematic Internet use (PIU). Few studies specifically addressed the boredom and loneliness dimensions in mediating the association between depression, anxiety, and stress levels and the onset of PIU. A nationwide population-based cross-sectional case-control study was carried out by recruiting a sample of Italian young people (aged 18–35). Only 1643 participants were selected for the analyses based on the age and the presence versus absence of PIU. Participants were mainly females (68.7%), with a mean age of 21.8 (SD = 1.7). Non-PIU individuals had significantly stable relationships (*p* = 0.012), siblings (*p* = 0.044) and lived with their family (*p* = 0.010), compared to PIU. PIU individuals displayed significantly higher depression, anxiety, and stress, as well as higher loneliness and boredom levels (all *p* < 0.001), compared to non-PIU. Depressive symptomatology predicted PIU and that their interaction is positively double mediated by boredom and loneliness (ß = 0.3829 (0.0245), 95%CI = 0.3349–0.4309). Our findings suggested that boredom and loneliness dimensions could act as mediators in the association between depressive symptomatology and the likelihood of PIU onset and maintenance.

## 1. Introduction

In recent years, there has been a progressive digitizalitation of our society, resulting in an intensification of technology use by the general population for daily life activities [1]. Indeed, Italy significantly increased its level of digitalization and information technology (IT) resources in 2019, and particularly following the COVID-19 pandemic [1,2,3], even though Italy currently ranks only in the 18th position regarding the digitizalitation level across all European countries, according to the Digital Economy and Society Index [2].

However, despite the great advantages in the improvement of the quality of life, determined by the digitally-based implementation of the daily activities, there has been indeed a progressive increase in the pathological use of technological tools, which facilitated the emergence of problematic/pathological Internet use (PIU) and Internet addiction (IA) [4,5]. PIU, firstly described in 1996, is characterized by uncontrolled and excessive Internet use [6]. In particular, the pathological threshold for diagnosing PIU consists of spending more than 6 h daily online in association with an individual’s impairment of daily life activities, due to the loss of control over one’s own behavior which becomes secondary to the Internet use [7]. Indeed, PIU currently represents an “umbrella” term including any typology of use of technological instruments through the Internet, including Internet gaming, online video streaming, social media use, and so forth [4,8]. Nowadays, despite the extreme methodological heterogeneity due to the different administered assessment tools and diagnostic criteria across different studies, a recent meta-analysis reported a worldwide PIU prevalence of around 7% [9]. In Europe, the PIU prevalence ranged from 2% in Norway to 18.3% in the UK, 13.3% in Hungary, 17.7% in Turkey, 15.7% in Germany [10,11,12,13,14]. In Italy, the PIU prevalence was variably reported due to the different inclusion/exclusion criteria adopted in the recruitment strategies, ranging from 0.8% to 22.1% [11,15,16,17,18]. In our previous study, a PIU prevalence rate of 23.3% was observed in an Italian sample of university students [19].

Furthermore, PIU has been also associated with various psychiatric disorders, particularly anxiety, depression, autism spectrum disorder, personality trait, Attention Deficit and Hyperactivity Disorder (ADHD), and psychosomatic diseases [5,20,21,22,23,24,25,26]. Recently, new psychopathological dimensions have been further investigated and considered in the onset, development, and maintenance of psychological distress and distress tolerance, as well as supposed to be implicated in the techno-addictions, such as proneness to loneliness and boredom [27,28,29,30,31,32,33]. Indeed, the diffusion of loneliness has been even defined as the current “modern behavioral epidemic”, due to its rapid and capillary spread among youngsters and the general population [34]. As with loneliness, boredom has also been found to be characterized by an increasing trend in the last decade (2008–2017), particularly among adolescents [35]. Boredom has been found to be associated with several psychiatric disorders, including depressive disorders, smartphone addiction, mobile social media, and subjective wellbeing, as well as substance and/or alcohol use disorder [35,36,37,38]. Boredom proneness can generate negative emotions, which may lead to depression, anxiety, loneliness, and lower levels of subjective wellbeing [32]. Moreover, boredom proneness may also act as a predictor of the overdependence on a certain object (and, hence, technology) [32]. Loneliness may favor the likelihood in developing depressive and anxiety disorders, alcohol and/or substance use and abuse, as well as incentivizing smoking habits [39]. Both these dimensions have been further investigated due to their dramatic increase among young people, particularly during the COVID-19 pandemic [38,40,41,42,43,44]. Moreover, recent studies also explored the potential association between the boredom and loneliness dimensions with PIU, suggesting a hypothetical mediatory role of both dimensions in the onset and maintenance of PIU in vulnerable people, or suggesting the moderated role of distress tolerance in the association between PIU and boredom proneness/loneliness [36], even though there are not currently studies specifically investigating both dimensions together in PIU in Italian young adults [27,28,29,30,31,32,33].

Therefore, considering that both dimensions have not been deeply investigated in their association between depressive, anxiety, and stress levels and the onset and/or maintenance of PIU in Italian young people, the current study aimed at: (a) measuring the prevalence of loneliness and boredom dimensions in a cohort of Italian young people (aged 18–24 years) with a diagnosis of PIU, by comparing them with an age- and sex-matched group of Italian young people (aged 18–24 years-old) without a diagnosis of PIU; (b) examining the association (if any) between anxiety, depressive, and/or stress levels and PIU, by particularly evaluating whether the relationship could be explained by a double mediatory role of boredom and loneliness dimensions.

## 2. Materials and Methods

### 2.1. Study Design and Recruitment Strategies

The current nationwide population-based cross-sectional case-control study was carried out by recruiting a sample of Italian young people (aged 18–35), using a snowball sampling strategy, during the following time frames from 25 January 2021 to 26 February 2021 and from 2 December 2021 to 23 February 2022. Participation was anonymous and voluntary without monetary or other incentives. All participants gave informed consent to take part in the study. Sample size was calculated using the WHO sample size calculator [45]. By keeping the values of confidence level as 99%, anticipated population proportion 0.5, an α error of 0.05, a power of 80%, and taking into consideration all variables to be entered in the multivariable analysis, a minimum sample size of 400 was *a priori* established to be reached for the present study, in order to obtain at least an effect size of >0.6. An ad hoc case report form (CRF) was developed to collect respondents’ socio-demographic and clinical characteristics. Participants were requested to fill out a set of self-report questionnaires administered online. The total sample, including 2304 subjects, was extrapolated as a post hoc analysis coming from the database of the SWATCH (Social Withdrawal And TeCno-mediated mental Health issues) study, aimed at investigating the main psychopathological determinants of the severe youth social withdrawal condition (hikikomori-like) and web-based psychopathologies in Italian adolescents/young adults. After data cleaning, only 1643 participants were selected for the analysis based on the age (only young adults aged 18–24) and the presence versus absence of PIU/non-PIU. The local Institutional Review Board approved the study. The study was conducted in accordance with the ethical principles outlined in the Declaration of Helsinki and according to the guidelines for Good Clinical Practice (GCP) (WHO, 2013). The CHERRIES checklist was followed in reporting the main findings of the present study [46].

### 2.2. Measurements

CRF collected a set of socio-demographic and clinical variables, including participants’ age, sex, relationship status, region, residence, socioeconomic status, the number of siblings, parents’ marital status, study years, current and/or past work activity, any psychological and/or psychiatric issues, and any past and/or current psychological and/or psychiatric contacts.

The Italian version of the Internet Addiction Test (IAT) [47] is a 20-item on a 5-point Likert scale assessing the presence and severity of compulsive use of the Internet, including compulsivity, escapism, and dependency. The scale considers PIU as an impulse-control disorder, by including all types of online activities. The IAT total score is the sum of the ratings given for each item. A cutoff of 50 was used to discriminate between PIU (IAT total score ≥ 50) and non-PIU (IAT total score < 50), consistent with previous studies carried out in Italian and international samples [7,19,48,49,50]. IAT includes also the following subscales: (a) salience (i.e., feelings of worries about the Internet, hides the behavior from others, use of Internet as a mental escape from distributing thoughts; feelings that life without the Internet would be boring, empty or joyless); (b) excessive use (i.e., excessive online behavior and compulsive usage); (c) neglect of work (i.e., Internet is experienced as a necessary appliance akin to the television, microwave or telephone); (d) anticipation (i.e., the subjects may feel compelled to use the Internet when offline); (e) lack of control (i.e., trouble managing his/her online time, frequently stays online longer than intended, others may complain about the amount of time he/she spends online); (f) neglect of social life (i.e., online relationships to cope with situational problems and/or to reduce mental tension and stress which also represents a measure of social quality of life). The Italian version of the IAT displayed good basic psychometric parameters of reliability, discriminant, and convergent validity [47]. The Cronbach’s α of the IAT in our study showed a good internal reliability (α = 0.898).

The Italian Loneliness Scale (ILS) [51] is a 20-item on a 5-point Likert scale assessing the subjective level of perceived loneliness. ILS is divided into the following three subscales: (a) emotional loneliness (6 items based on emotional abandonment and lack of companionship); (b) social loneliness (5 items assessing feelings of sociability and the presence of meaningful relationships), and (c) general level of isolation (7 items focusing on feelings of isolation). The ILS displayed good basic psychometric parameters of reliability, discriminant, and convergent validity [51]. The Cronbach’s α of the ILS in our study showed an excellent internal reliability (α = 0.911).

The Italian validation of the Multidimensional State Boredom Scale (MSBS) [52] assessed the boredom construct which includes: (a) the disconnection or struggle to engage in one’s surrounding environment; (b) boredom as a negative or undesirable experience; (c) emotional and cognitive experiences accompanying the boredom; (d) changes in time perception; (e) coping with boredom, and (f) not being bored. MSBS is a 29-item on a 7-point Likert scale. The scale provides a total boredom score (determined by the sum of all items) and five subscales. The subscales measures the following five boredom components: (a) disengagement (i.e., the measure of the lack of engagement); (b) high arousal (i.e., the measure of the presence of restlessness, agitation, and frustration symptomatology related to the boredom experience); (c) low arousal (i.e., the measure on how much the subject experiences their own environment as redundant, monotonous, with low stimuli, or meaningless); (d) inattention (i.e., the measure on how much the subject finds difficulty in focusing their attention); (e) time perception (i.e., the measure on how the subject perceives how time flows). The MSBS displayed good basic psychometric parameters of reliability, discriminant, and convergent validity [52]. The Cronbach’s α of the MSBS in our study showed an excellent internal reliability (α = 0.921).

The Italian version of the Depression, Anxiety and Stress Scale-21 (DASS-21) [53] is a 21-item self-reported questionnaire consisting of 7 items for each of the three subscales (depression, anxiety, and stress). Every item was evaluated on a scale from 0 (did not apply to me at all) to 3 (applied to me very much). The total score assesses the general distress dimension. Total score is obtained by adding up the scores on the items per (sub) scale and multiplying them by a factor of 2. The total score ranges from 0–120. Total score for each of the subscales may range between 0 and 42, with 5 levels of severity with different ranges depending on three subscales [53]. The depression subscale assesses dysphoria, hopelessness, devaluation of life, self-deprecation, and lack of interest/involvement, anhedonia, and inertia. The anxiety subscale assesses autonomic arousal, skeletal muscle effects, situational anxiety, and subjective experience of anxious affect. The stress scale is sensitive to levels of chronic non-specific arousal and it assesses difficulty in relaxing, nervous arousal, and being easily upset/agitated, irritable/over-reactive, and impatient. The Cronbach’s α of the DASS-21 in our study showed an excellent internal reliability (α = 0.947).

### 2.3. Statistical Analysis

Descriptive statistics were performed in order to describe the socio-demographic and clinical characteristics of the sample. Categorical variables are summarized as frequency (n) and percentage (%) whilst continuous variables as means (standard deviation (SD)). After analyzing the continuous variables for skewness, kurtosis, normality distribution through the Kolmogorov-Smirnov test, and the equality of variances by Levene test, parametric or non-parametric statistical tests were used when appropriate. Categorical variables were compared using the χ^2^ test as well as all socio-demographic variables between two groups (PIU versus non-PIU). Since our continuous variables showed a normal distribution, independent samples Student’s T-test and two-way tailored analysis of variance (ANOVA) were used to investigate the level of severity of PIU (as measured by IAT), according to a set of categorical socio-demographic and psychopathological independent variables (i.e., educational levels, parental affective status, etc.). Bivariate Pearson’s correlations have been used to investigate potential relationships between IAT total score and the following continuous variables: DASS total score, DASS depressive symptoms, DASS anxiety symptoms, DASS stress symptoms, ILS total score and subscales, and MSBS total score and subscales. Statistical analyses were firstly carried out by considering PIU (as measured by IAT) as a primary outcome in order to evaluate whether loneliness (as measured by ILS) and boredom (as measured by MSBS) dimensions could be predictors of a higher risk of developing PIU in our cohort of young people. PIU was firstly evaluated as a continuous variable and then was categorized as dichotomous (PIU versus non-PIU). Therefore, in order to identify possible predictors of the levels of PIU (as measured by IAT), a multivariate linear regression model with a Bonferroni’s adjustment for multiple comparison tests was performed, including as independent variables: DASS-21 total score, DASS-21 depression, anxiety, and stress subscales, sex, ILS total and subscales, and MSBS total and subscales. In addition, a stepwise binary logistic regression analysis, with a Bonferroni’s adjustment for multiple comparison tests, was carried out in order to evaluate the predictors associated with the presence of PIU (vs. non-PIU) after categorizing IAT total score into two dichotomous values according to the established cut-off of 50. The estimated odds ratios (OR) along with the 95% of confidence intervals (95% CI), and standardized coefficient β values were generated for each variable. Following the identification of significant predictors of PIU, PROCESS macro (version 4.1, April 2022) for SPSS (IBM SPSS Statistics, Chicago, IL, USA) was run to carry out mediation analyses (Model 6) to test whether the direct or indirect effect of depression and/or anxiety and/or stress levels (as independent variables) on PIU (as dependent variable), were mediated by the boredom and/or loneliness dimensions. Indicators of indirect effects were tested using a bias-corrected bootstrapping (n = 5000) with 95% CI, by setting a statistical significance when the 95% CI does not contain zero. For all analyses, the level of statistical significance was set at *p* < 0.05, two-tailed. All statistical analyses were performed using the software Statistical Package for Social Science (SPSS) version 27.0 for MacOS (IBM SPSS Statistics, Chicago, IL, USA).

## 3. Results

### 3.1. Socio-Demographic and Psychopathological Characteristics of the Sample

The final sample included in the present post hoc analysis was of 1643 young people. Participants were mostly aged 20–24 (88.5%; n = 1454), without sex-based differences (*p* = 0.145). The mean years of educational level was 16.2 (SD = 1.7). Table 1 summarizes all socio-demographic features of the sample. Overall, the sample displayed a severe general psychopathology in 49.4% of the participants, particularly, most participants showed a severe-to-extremely-severe depressive symptomatology (49.4%; n = 813), a severe-to-extremely-severe anxiety symptomatology (49.0%; n = 804), and severe-to-extremely-severe stress symptomatology (48.6%; n = 800) (Table 2).

The mean total score at IAT was 41.5 (SD = 12.6), IAT salience subscale was 9.3 (SD = 3.5), IAT excessive use was 11.1 (SD = 3.7), IAT neglect work was 6.8 (SD = 2.8), IAT Anticipation was 4.3 (SD = 1.7), IAT lack of control was 6.8 (SD = 2.7), and IAT neglect social life was 3.2 (SD = 1.3) (Table 3). No significant sex-based differences were found at IAT total score (*p* = 0.610). Significant sex-based differences were found for IAT lack of control and IAT neglect social life subscales (respectively, *p* = 0.002 and *p* < 0.001), with females reporting higher levels at IAT lack of control while males were reporting significantly higher scores at neglect social life, compared to respective counterparts. No significant differences were found regarding other investigated socio-demographic features, except for the current marital status being those with an unstable affective relationship, those who displayed significantly higher IAT total scores compared to other groups (F(31639) = 6.522, *p* < 0.001). Among the total sample, 23.3% of participants were classified as having PIU (n = 382). No significant age-based (*p* = 0.864) and sex-based (*p* = 0.399) differences were found between PIU versus non-PIU groups regarding the educational levels (*p* = 0.865) (Table 1).

The mean total score at MSBS was 121.5 (SD = 42.3), MSBS disengagement subscale was 44.6 (SD = 15.9), MSBS high arousal subscale was 20.2 (SD = 8.5), MSBS inattention subscale was 19.7 (SD = 6.7), MSBS low arousal subscale was 21.3 (SD = 9.2), and MSBS time perception subscale was 15.7 (SD = 9.5) (Table 3). Most of the participants displayed an MSBS total score significant for boredom dimension (above 88), being represented by around 76.8% of the sample (n = 1262), without significant sex-based differences (*p* = 0.121). No significant differences were found in MSBS scores depending on other participants’ socio-demographic features.

The mean total score at ILS was 47.4 (SD = 12.7), ILS social loneliness was 10.5 (SD = 4.1), ILS emotional loneliness was 14.9 (SD = 4.3), and ILS general loneliness was 16.0 (SD = 5.7) (Table 3). Significant sex-based differences were found only for the ILS social loneliness subscale (*p* = 0.002). Single participants reported significantly higher ILS total and subscale scores compared to other affective conditions (all with *p* < 0.001).

### 3.2. Socio-Demographic and Clinical Predictors of PIU Versus Non-PIU

Non-PIU individuals who significantly displayed higher rates of a stable relationship (*p* = 0.012), more frequently declared to live together with their nuclear family (*p* = 0.010) and have siblings (*p* = 0.044), compared to PIU individuals (Table 1). PIU individuals displayed significantly higher levels of depression, anxiety, and stress levels, compared to non-PIU (all with *p* < 0.001) (Table 2). Furthermore, significantly higher levels of loneliness were reported among PIU individuals compared to non-PIU, also in all ILS subscales (all with *p* < 0.001) (Table 2). Similarly, higher levels of boredom dimension and all MSBS subscales were found in PIU compared to non-PIU young people (all with *p* < 0.001) (Table 2). All correlations between IAT total scores and subscales and all continuous variables under investigation are summarized in the Supplementary Materials.

According to the multivariate regression model, PIU levels were negatively predicted by MSBS low arousal levels (Beta coefficient, B = −0.386; 95%Confidence Interval, CI = (−0.523)–(−0.249); *p* < 0.001) and MSBS time perception (B = −0.083; 95%CI = (−0.149)–(−0.016); *p* = 0.015). While PIU levels were positively predicted by MSBS inattention (B = 0.695; 95%CI = 0.574–0.815; *p* < 0.001), MSBS disengagement (B = 0.154; 95%CI = 0.078–0.230; *p* < 0.001), ILS total (B = 0.184; 95%CI = 0.082–0.286; *p* < 0.001), ILS emotional loneliness (B = 0.279; 95%CI = 0.011–0.548; *p* = 0.041), and depressive levels (B = 0.124; 95%CI = 0.056–0.192; *p* < 0.001). These variables statistically significantly predicted problematic Internet use (F(8, 1634) = 71.149, *p* < 0.001, R^2^ = 0.258) (Table 4). A logistic regression analysis was performed to ascertain the effects of loneliness, boredom, and depression on the likelihood of developing PIU. The logistic regression model was statistically significant, χ^2^(4) = 259.947, *p* < 0.001. The model explained 22.1% (Nagelkerke R^2^) of the variance in PIU and correctly classified 77.7% of cases. According to the logistic regression model, PIU was significantly predicted by higher levels at MSBS inattention, ILS total scores, and depressive levels while it was negatively predicted by higher levels at MSBS low arousal subscale.

**Figure 1:**
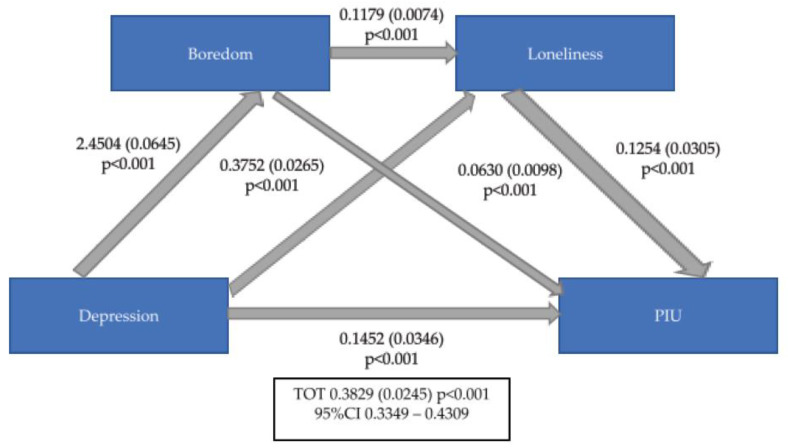
Mediation role of Boredom and Loneliness between Depression and PIU. PIU: Problematic Use of Internet; TOT: Total; CI: Confidence Interval.

**Figure 2:**
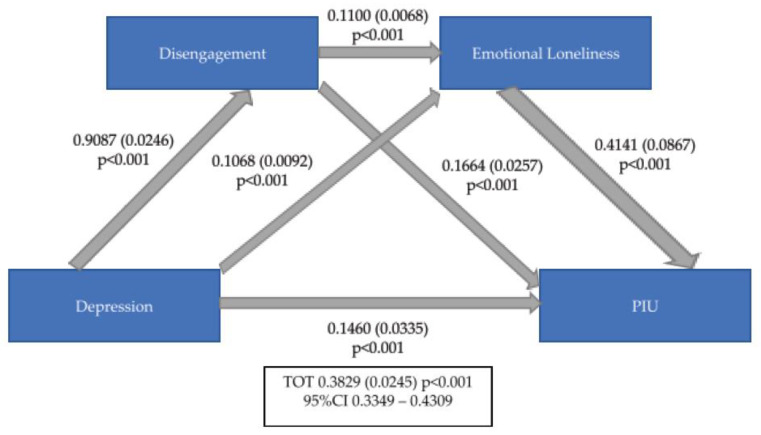
Mediation role of Disengagement Boredom and Emotional Loneliness between Depression and PIU. PIU: Problematic Use of Internet; TOT: Total; CI: Confidence Interval.

**Figure 3:**
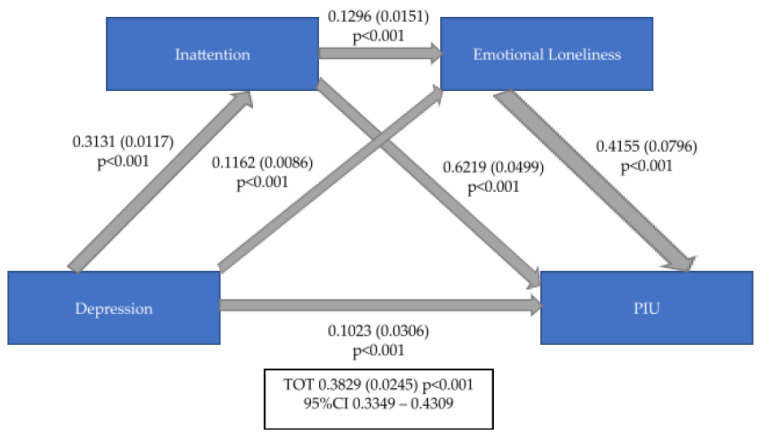
Mediation role of Inattention Boredom and Emotional Loneliness between Depression and PIU. PIU: Problematic Use of Internet; TOT: Total; CI: Confidence Interval.

Mediation analyses showed that depressive symptomatology (as measured by DASS-21 depression subscale) predicted PIU (as measured by IAT) and that their interaction is double mediated by MSBS and ILS total scores (Figure 1), and, particularly, is also double mediated by MSBS disengagement subscale and ILS emotional loneliness subscales (Figure 2) as well as by MSBS inattention subscale and ILS emotional loneliness subscales (Figure 3) (ß = 0.3829 (0.0245), 95%CI = 0.3349–0.4309).

## 4. Discussion

Our findings reported that boredom and loneliness dimensions act as double mediators in the association between depressive symptomatology and the likelihood of PIU onset and maintenance. In particular, mediation analyses showed that depressive symptomatology predicted PIU and that their interaction is double mediated by the overall boredom and loneliness dimensions, particularly by the disengagement component of the boredom dimension and the emotional loneliness as well as by the inattention component of the boredom dimension and emotional loneliness. Overall, PIU individuals significantly reported higher levels of boredom and loneliness dimensions, compared to non-PIU individuals, by suggesting a potential relationship between both dimensions and the PIU onset and/or maintenance. According to our findings, PIU seemed to be negatively predicted by MSBS low arousal levels (which indicated all experiences and behaviours more specifically related to internalizing aspects) and MSBS time perception levels (which describes the perceptions and experiences related to the slow passage of the time). Contrarily, PIU appeared to be positively predicted by higher MSBS inattention (which indicated the difficulty in focusing attention on events and/or activities and/or interests) and disengagement levels (which indicated the perceived lack of an individual’s involvement in any activities). Furthermore, our findings reported that higher ILS emotional loneliness appeared to significantly predict PIU onset and maintenance. Emotional loneliness indicates an unpleasant feeling resulting from the perception that is missing from an intimate attachment relationship or that the existing relationship is inadequate [51].

Despite the cross-sectional nature of the research design, it may limit the generalizability of the present findings as it does not allow to draw a causal relationship between boredom proneness and loneliness dimensions and PIU; many hypotheses have been considered here and discussed. In our findings, boredom proneness represents a predictor for the high risk of PIU development and a mediating variable between depressive symptomatology and PIU in youths, as already confirmed by previous studies [32,33,54,55]. Boredom is described as a state of relatively low arousal and dissatisfaction, which is attributed to an insufficient environmental stimulation and an unsuccessful individual’s engagement in enjoyable activities [56,57]. Higher levels of boredom proneness have been also associated with poorly sustained attention, a predisposing trait accompanied by low arousal to internal and external stimuli and difficulty in concentrating [58,59,60]. Overall, boredom represents a dimension strictly associated with negative affects, behaviors, interpersonal relationships, and occupational situations; as well, boredom has been demonstrated to be accompanied by higher sensitivity for rewarding behaviors [54,61,62,63,64,65]. Within this context, hence, boredom feelings should increase the likelihood that individuals disengage from their current goals and engage in new activities (not necessarily functional/useful in that moment) [66]. Within this context, Internet use is able to provide rapid reactions, immediate rewards, and multiple opportunities for performing several and different activities which may in turn reduce the boredom feelings, even though it has been supposed that high boredom levels could be more likely associated to the development and maintenance of PIU [67], according to the compensatory Internet use theory (CIUT) [68]. According to the CIUT, the lack of social stimulation in real life may predispose individuals to boredom proneness and the use of the Internet tool as a socializing instrument for relieving boredom feelings and loneliness. Moreover, it has been also hypothesized that boredom may act as a ‘stop emotion’ able to trigger disengagement from the current task and more likely facilitate the individual’s interaction with technological tools [69]. Furthermore, in our findings, the loneliness dimension, particularly emotional loneliness, seemed to cover a pivotal role as a mediatory factor in the relationship between depression and the emergence of PIU. Loneliness (i.e., a perceived deficit in social connection and relationships) represents a critical determinant of wellbeing and could be associated with higher rates of anxiety, depression, and suicidality [70,71]. Furthermore, the confirmed association between depressive symptomatology and the presence of PIU, as already documented in the literature [72,73,74], was also present during the COVID-19 pandemic in which social isolation and loneliness may have accelerated the onset of depressive states [26,75], and should also be considered and interpreted by a clinical point of view. In fact, this association should guide clinicians towards an early assessment and prevention strategy, particularly in those of a more vulnerable younger population who may present those predisposing/risk factors for loneliness and depressive status as well as for those individuals with boredom proneness traits who may be more sensible to the development/shift towards PIU and/or other problematic use of technological tools. Therefore, preventive strategies should be performed and structured in order to early diagnose high-risk individuals, in order to implement early interventions which could modify the psychopathological derive. In addition, as many studies on the neurobiological underpinnings of PIU have been recently developed demonstrating how technology may determine structural and functional brain changes [76,77,78,79], one could argue how it could be interesting to evaluate how boredom and loneliness dimensions could differently be associated with specific structural and functional brain areas in PIU subjects, compared to non-PIU subjects or other addictions. In fact, in PIU subjects, brain alterations have been described, such as white matter integrity of the corpus callosum [76], an increased gray matter volume of orbitofrontal cortex [77], striatum [78], anterior cingulate cortex, dorsolateral prefrontal cortex, and supplementary motor area [79].

However, several limitations should be also considered in the present study. Firstly, the cross-sectional nature of the design study may limit the generalizability about the causal relationship between boredom and loneliness dimensions and the onset of PIU. Secondly, the sample was recruited by using a snowball recruitment strategy online which could determine selection and recruitment biases. Thirdly, the current study did not assess the boredom and loneliness levels before the emergence of PIU and of the depressive symptomatology, hence, it is not possible to draw definitive conclusions regarding the association between a predisposing level of the boredom and loneliness dimensions in favoring the emergence and/or maintenance of PIU among those vulnerable depressive subjects. Fourthly, since most data collected in the present study came from self-reported tools, this might determine potential social desirability biases. Fifthly, it could be helpful to draw further research hypotheses to associate the measure of these clinical biomarkers associated with PIU with those derived by neuroimaging studies, which could more deeply investigate the neurobiological underpinnings of PIU mediated by boredom and loneliness dimensions, compared to PIU mediated by other psychopathological determinants.

## 5. Conclusions

Overall, the present findings clearly suggest the need to adequately and early identify those at-risk young people manifesting clinically significant boredom and/or loneliness levels in order to preventively modify the psychopathological trajectory towards the emergence of a problematic use of technological tools, including PIU. Furthermore, depressive young people could represent a vulnerable population who should be carefully evaluated regarding their boredom and social loneliness dimensions which could favor an onset of PIU. Moreover, ad hoc specific treatment and educational tools should be implemented in youth depression in order to stimulate them towards attractive and creative activities with the aim to reduce boredom levels as well as incentivizing socializing activities (i.e., social skills training, enhancing social support, increasing opportunities for social interaction, addressing maladaptive social cognition) to attenuate loneliness feelings. Emotional regulation, impulse control, and motivation-based interventions in both boredom proneness as well as depressive young people with clinically significant boredom and/or emotional loneliness levels should be implemented as preventive and treatment strategies to reduce the risk of PIU onset and maintenance. Further longitudinal studies addressed specifically to clinical samples should be implemented in order to clearly investigate the causal relationship between PIU onset and depressive symptomatology, considering the variable levels of boredom and loneliness dimensions as well as considering how and which interventions addressed to boredom and loneliness levels could significantly reduce depressive symptomatology in PIU individuals and/or reduce PIU by improving boredom and loneliness dimensions.

## Figures and Tables

**Table 1:** Socio-demographic characteristics of the sample.

	Total Sample	Not-PIU	PIU	*p*-Value
**Sex**				
Males	514 (31.3%)	392 (31.3%)	122 (31.9%)	χ^2^ = 0.099 *p* = 0.753
Females	1129 (68.7%)	869 (68.9%)	260 (68.1%)	
**Parental legal status**				
Married	1298 (79%)	1001 (79.4%)	297 (77.7%)	χ^2^ = 1.851 *p* = 0.763
Unmarried parents	22 (1.3%)	18 (1.4%)	4 (1.0%)	
Separated	121 (7.4%)	94 (7.5%)	27 (7.1%)	
Divorced	139 (8.5%)	102 (8.1%)	37 (9.7%)	
Widowed	63 (3.8%)	46 (3.6%)	17 (4.5%)	
**Living condition**				
With their nuclear family	1025 (62.3%)	787 (62.4%)	238 (62.3%)	χ^2^ = 3.404 *p* = 0.845
With one of their parents	156 (9.5%)	119 (9.4%)	37 (9.7%)	
With other relatives (not parents)	15 (0.9%)	14 (1.1%)	1 (0.3%)	
Alone	65 (4.0%)	48 (3.8%)	17 (4.5%)	
In a university hostel/boarding school	42 (2.6%)	34 (2.7%)	8 (2.1%)	
Together with other university classmates	249 (15.2%)	188 (14.9%)	61 (16.0%)	
With their partner	48 (2.9%)	37 (2.9%)	11 (2.9%)	
Other	43 (2.9%)	34 (2.7%)	9 (2.4%)	
**Personal psychological distress history**				
None	420 (25.6%)	359 (28.5%)	61 (16.0%)	χ^2^ = 25.207 ***p* < 0.001**
Yes, without professional support	658 (41.7%)	513 (40.7%)	172 (25.1%)	
Yes, with professional support	538 (32.7%)	389 (30.8%)	149 (39.0%)	
**Siblings**				
Yes	1347 (82.0%)	1039 (82.4%)	308 (80.6%)	χ^2^ = 0.620 *p* = 0.431
No	296 (18.0%)	222 (17.6%)	74 (19.4%)	
**Relationship status**				
Single	794 (48.3%)	585 (46.4%)	209 (54.7%)	χ^2^ = 8.128 ***p* = 0.004**
In a relationship	849 (51.7%)	676 (53.6%)	173 (45.3%)	

In **bold** are the significant *p*-values.

**Table 2:** Clinical characteristics of the sample.

	Total Sample	Not-PIU	PIU	*p*-Value
**DASS-21 Depression Subscale**				
Normal	548 (33.4%)	495 (39.3%)	53 (13.9%)	χ^2^ = 143.353 ***p* < 0.001**
Mild	179 (10.9%)	37 (11.5%)	34 (8.9%)	
Moderate	281 (17.1%)	74 (16.4%)	74 (19.4%)	
Severe	310 (18.9%)	76 (18.6%)	76 (19.9%)	
Extremely Severe	325 (19.8%)	145 (14.3%)	145 (38.0%)	
**DASS-21 Anxiety Subscale**				
Normal	839 (51.1%)	708 (56.1%)	131 (34.3%)	χ^2^ = 64.454 ***p* < 0.001**
Mild	187 (11.4%)	125 (9.9%)	62 (16.2%)	
Moderate	248 (15.1%)	183 (14.5%)	65 (17.0%)	
Severe	224 (13.6%)	156 (12.4%)	68 (17.8%)	
Extremely Severe	145 (8.8%)	89 (7.1%)	56 (14.7%)	
**DASS-21 Stress Subscale**				
Normal	358 (21.8%)	324 (25.7%)	34 (8.9%)	χ^2^ = 86.189 ***p* < 0.001**
Mild	172 (10.5%)	141 (11.2%)	31 (8.1%)	
Moderate	313 (19.1%)	246 (19.5%)	67 (17.5%)	
Severe	451 (27.4%)	334 (26.5%)	117 (30.6%)	
Extremely Severe	349 (21.2%)	216 (17.1%)	133 (23.3%)	

DASS-21: Depression, Anxiety and Stress Scale-21. In **bold** are the significant *p*-values.

**Table 3:** Psychopathological features of the sample.

	Total Sample	Not-PIU	PIU	*p*-Value
**DASS-21 Depression subscale**	21.2 (11.8)	19.3 (11.5)	27.6 (10.4)	t = −13.303 ***p* < 0.001**
**DASS-21 Anxiety subscale**	16.1 (11.0)	15.0 (10.7)	19.8 (10.8)	t = −7.849 ***p* < 0.001**
**DASS-21 Stress subscale**	23.8 (10.5)	22.5 (10.4)	28.3 (9.4)	t = −10.399 ***p* < 0.001**
**DASS-21 Total score**	61.1 (30.0)	56.7 (29.3)	75.8 (27.0)	t = −11.848 ***p* < 0.001**
**ILS Social Loneliness**	10.4 (4.1)	10.1 (4.0)	11.7 (4.1)	t = −7.115 ***p* < 0.001**
**ILS Emotional Loneliness**	15.0 (4.2)	14.4 (4.2)	16.9 (4.0)	t = −10.542 ***p* < 0.001**
**ILS General Loneliness**	16 (5.7)	15.2 (5.5)	18.5 (5.4)	t = −10.221 ***p* < 0.001**
**ILS Total score**	47.4 (12.6)	45.6 (12.3)	53.5 (11.8)	t = −11.101 ***p* < 0.001**
**MSBS Disengagement**	44.6 (16.0)	42.0 (15.9)	53.1 (13.0)	t = −13.858 ***p* < 0.001**
**MSBS High Arousal**	20.2 (8.4)	19.0 (8.4)	24.4 (7.3)	t = −12.356 ***p* < 0.001**
**MSBS Inattention**	19.7 (6.7)	18.5 (6.8)	23.6 (4.6)	t = −17.098 ***p* < 0.001**
**MSBS Low Arousal**	21.3 (9.2)	20.0 (9.1)	25.6 (8.0)	t = −11.477 ***p* < 0.001**
**MSBS Time Perception**	15.7 (9.5)	15.1 (9.3)	17.5 (10.0)	t = −4.202 ***p* < 0.001**
**MSBS Total Score**	121.5 (42.3)	114.6 (42.1)	144.2 (34.2)	t = −14.004 ***p* < 0.001**

DASS-21: Depression, Anxiety and Stress Scale-21; ILS: Italian Loneliness Scale; MSBS: Multidimensional State Boredom Scale; IAT: Internet Addiction Scale. In **bold** are the significant *p*-values.

**Table 4:** Multiple Linear Regression with IAT total score.

	B	SE	β	t	*p*-Value	95%IC Lower Limit	95%IC Upper Limit
**MSBS Inattention**	0.694	0.061	0.371	11.313	**<0.001**	0.574	0.815
**ILS Total Score**	0.184	0.052	0.186	3.538	**<0.001**	0.082	0.286
**MSBS Low Arousal**	−0.386	0.070	−0.283	−5.525	**<0.001**	−0.523	−0.249
**DASS-21 Depression Subscale**	0.124	0.035	0.117	3.590	**<0.001**	0.056	0.192
**MSBS Disengagement**	0.154	0.039	0.195	3.989	**<0.001**	0.078	0.230
**MSBS Time Perception**	−0.083	0.034	−0.062	−2.442	**0.015**	−0.149	−0.016
**ILS Emotional Loneliness**	0.279	0.137	0.095	2.041	**0.041**	0.011	0.548

SE: Standard Error; DASS-21: Depression. Anxiety and Stress Scale-21; ILS: Italian Loneliness Scale; MSBS: Multidimensional State Boredom Scale. In **bold** are the significant *p*-values.

## Data Availability

The data presented in this study are available on request from the corresponding author.

## Supplementary Materials

The following supporting information can be downloaded at: https://www.mdpi.com/article/10.3390/ijerph20054446/s1.
