# Supplementary material for: The Mediatory Role of the Boredom and Loneliness Dimensions in the Development of Problematic Internet Use
Source: Int J Environ Res Public Health. 2023 Mar 2;20(5):4446. doi: 10.3390/ijerph20054446 (PMC10001960; doi:10.3390/ijerph20054446)
Supplement: Supplementary file 1 [file ijerph-20-04446-s001.zip › ijerph-2169338-supplementary.pdf]

Supplementary File. Correlations between psychopathological variables.

	IAT Subscale Salience	IAT Subscale Excessive	IAT Subscale Neglect	IAT Subscale Anticipati	IAT Subscale Lack of	IAT Subscale Neglect	IAT Total score	MSBS Disengag ement	MSBS High Arousal	MSBS Inattentio n	MSBS Low Arousal	MSBS Time Perceptio	MSBS Total score	ILS Social Lonelines	ILS Emotiona l	ILS General Lonelines	ILS Total score	DASS-21 Depressio n	DASS-21 Anxious Subscale	DASS-21 Stress Subscale
IAT Subscale Excessive Use	0,668																			
IAT Subscale Neglect Work	0,558	0,667																		
IAT Subscale Anticipation	0,519	0,473	0,411																	
IAT Subscale Lack of Controll	0,569	0,738	0,636	0,433																
IAT Subscale Neglect Social Life	0,435	0,379	0,313	0,31	0,291															
IAT Total Score	0,841	0,895	0,805	0,641	0,825	0,514														
MSBS Disengagement	0,374	0,343	0,345	0,199	0,285	0,164	0,39													
MSBS High Arousal	0,362	0,291	0,295	0,186	0,25	0,12	0,345	0,792												
MSBS Inattention	0,348	0,405	0,481	0,195	0,389	0,13	0,45	0,739	0,679											
MSBS Low Arousal	0,355	0,276	0,276	0,161	0,201	0,162	0,325	0,822	0,762	0,629										
MSBS Time Perception	0,172	0,101	0,101	0,06	0,076	0,012	0,127	0,521	0,504	0,375	0,433									
MSBS Total score	0,384	0,334	0,348	0,192	0,28	0,144	0,386	0,947	0,884	0,793	0,876	0,674								
ILS Social Loneliness	0,223	0,168	0,176	0,079	0,088	0,136	0,196	0,301	0,278	0,193	0,498	0,086	0,327							
ILS Emotional Loneliness	0,356	0,289	0,302	0,186	0,224	0,206	0,348	0,611	0,541	0,458	0,728	0,304	0,637	0,511						
ILS General Loneliness	0,336	0,272	0,287	0,159	0,193	0,18	0,321	0,572	0,519	0,419	0,753	0,224	0,599	0,662	0,791					
ILS Total score	0,361	0,291	0,307	0,173	0,209	0,201	0,346	0,601	0,549	0,444	0,785	0,253	0,634	0,781	0,881	0,949				
DASS-21 Depression Subscale	0,354	0,311	0,336	0,181	0,239	0,168	0,36	0,674	0,633	0,552	0,721	0,268	0,684	0,348	0,574	0,599	0,62			
DASS-21 Anxious Subscale	0,24	0,211	0,181	0,126	0,177	0,11	0,237	0,406	0,514	0,351	0,435	0,188	0,448	0,17	0,367	0,361	0,371	0,65		
DASS-21 Stress Subscale	0,298	0,261	0,258	0,185	0,226	0,11	0,304	0,547	0,656	0,477	0,544	0,242	0,585	0,192	0,455	0,439	0,458	0,765	0,734	
DASS-21 Total score	0,332	0,291	0,289	0,182	0,239	0,145	0,335	0,606	0,668	0,513	0,634	0,259	0,639	0,267	0,52	0,523	0,541	0,901	0,88	0,921

DASS-21: Depression. Anxiety and Stress Scale-21; ILS: Italian Loneliness Scale; MSBS: Multidimensional State Boredom Scale; IAT: Internet Addiction Scale.
